# Strength exercise weakens aerobic exercise-induced cognitive improvements in rats

**DOI:** 10.1371/journal.pone.0205562

**Published:** 2018-10-10

**Authors:** Yongsheng Lan, Zhaoyuan Huang, Yanjie Jiang, Xuehua Zhou, Jingyu Zhang, Dianyu Zhang, Bo Wang, Guangqing Hou

**Affiliations:** 1 College of Physical Education, Changchun Normal University, Changchun, Jilin, China; 2 Department of Orthopaedics, Tongji Hospital, Tongji Medical College, Huazhong University of Science and Technology, Wuhan, Hubei, China; University Hospital Wurzburg, GERMANY

## Abstract

Aerobic exercise improves cognitive function and adult hippocampal neurogenesis. However, the effects of aerobic exercise combined with strength exercise on cognitive function and adult hippocampal neurogenesis are still unknown. In this study, we established exercise paradigms in rats to mimic aerobic exercise combined with low- and high-intensity strength exercise. We found that aerobic exercise improved spatial learning and memory as well as adult hippocampal neurogenesis, whereas strength exercise suppressed aerobic exercise-induced cognitive improvements and adult hippocampal neurogenesis in an intensity-dependent manner. Furthermore, the levels of β-hydroxybutyrate (β-HB) and its downstream effector brain-derived neurotrophic factor (BDNF) were increased in the aerobic exercise group, and strength exercise impaired the aerobic exercise-induced increases in β-HB and BDNF mRNA levels. Taken together, these results demonstrated that strength exercise weakened aerobic exercise-induced cognitive improvements and adult hippocampal neurogenesis in rats.

## Introduction

Aerobic exercise is well known for its beneficial effects on cognitive performance [1–4]. Aerobic exercise refers to the use of aerobic metabolism to adequately meet energy demands during exercise. This process reflects the delivery of oxygen in the blood, which is pumped by the heart and transported to muscles. Increasing aerobic exercise ability means expanding the capacity of the heart and cardiovascular system to perform the task, supplying more oxygen and energy to the entire body, including the brain [5].

Adult hippocampal neurogenesis is a recently recognized form of brain plasticity that has been observed in many mammals, including humans [6]. The neural stem cells in the subgranular zone of the hippocampal dentate gyrus (DG) undergo a dynamic process, including proliferation, neuronal fate specification, neuronal migration, and synaptic integration [7]. These newly generated neurons in the DG contribute to memory formation [8] and spatial pattern separation [9], while reduced adult hippocampal neurogenesis contributes to stress-induced anxiety- and depressive-like behaviors [10]. Previous studies have shown that aerobic exercise increases adult hippocampal neurogenesis and improves cognitive function [1–4]. These effects of aerobic exercise are strongly associated with brain-derived neurotrophic factor (BDNF), which improves various aspects of adult hippocampal neurogenesis, such as neural stem cell proliferation, neuronal survival, dendritic arborization and synaptic plasticity [11–13]. Strength exercise, or resistance exercise, is primarily anaerobic exercise, which uses anaerobic glycolysis as the major source of power. Interestingly, strength exercise also increases BDNF levels in the serum [14]. However, whether aerobic exercise combined with strength exercise can further increase adult hippocampal neurogenesis is poorly understood.

To determine the effects of aerobic exercise combined with strength exercise on cognitive performance and adult hippocampal neurogenesis, we established four exercise paradigms in rats: aerobic exercise combined with low-intensity strength exercise, aerobic exercise combined with high-intensity strength exercise, aerobic exercise as a positive control and the sedentary condition as a negative control. We found that aerobic exercise improved cognitive performance and increased adult hippocampal neurogenesis, while aerobic exercise combined with low- or high-intensity strength exercise decreased these benefits. We further found that the reduction in these benefits may be caused by decreases in β-hydroxybutyrate (β**-**HB) levels and BDNF expression. Taken together, these results demonstrate that strength exercise negatively regulates aerobic exercise-induced cognitive improvements and adult hippocampal neurogenesis in rats.

## Materials and methods

### Animals

Forty-eight male Wistar rats, 8 weeks old, were housed in the premises of the animal research unit at Changchun Normal University. Food and water were freely available. The temperature and humidity were controlled at 22 ± 1°C and 50 ± 10%, respectively. The room was maintained on a 12:12 hour light:dark cycle. All procedures were conducted during the light portion of the cycle. Experiments with animals were approved by the Institutional Animal Care and Use Committee of Changchun Normal University.

### Familiarity training

The rats were randomly divided into one sedentary group (Sed, n = 12) and three exercise groups: an aerobic exercise group (AER, n = 12), an aerobic exercise + low-intensity strength exercise group (AER&LST, n = 12) and an aerobic exercise + high-intensity strength exercise group (AER&HST, n = 12). All rats in the exercise groups initially performed familiarity training for 1 week. During this period, the rats in the AER group were trained on a 0-degree inclination treadmill with an electrified grip at the end of each lane at a speed of 15 m/min, 5 times per week (from Monday to Friday). The exercise duration gradually increased from 15 minutes to 60 minutes, with a 15-minute increase per day. The rats in the AER&LST group and the AER&HST group performed the same protocol as the AER group on the first three days of familiarity training. On the 4^th^ day and the 5^th^ day, the rats in these two groups climbed on the 5-degree inclination uphill treadmill for 60 minutes with an extra load of 5% body weight and 10% body weight, respectively.

### Formal training

All formal training protocols were conducted on a treadmill, 5 times per week (from Monday to Friday) for 8 weeks, with 2 days of recovery (Saturday and Sunday). The rats were allowed to warm up for 5 minutes at a speed of 10 m/min before each training regimen. Each training regimen included two sessions and 5 minutes of rest. Further details about each training regimen are presented below.

The AER regimen contained two identical training sessions. The parameters of each training session were as follows: the running speed was 15 m/min; the inclination of the belt was 0 degrees; and the exercise duration was 30 minutes. There was a 5-minute rest between the two training sessions.

The AER&LST regimen included aerobic exercise sessions and low-intensity strength exercise sessions. The aerobic exercise session parameters were the same as those described for the AER regimen. However, in the low-intensity training session, a load pouch was fixed to the lower back of each rat as an extra load. The extra load was decided by the mean body weight of all rats in the AER&LST group, which was recorded each Monday. In this group, the extra load was 5% body weight. The treadmill inclination was kept at a constant 5 degrees uphill. The running speed was 15 m/min. The exercise duration was 30 minutes. There was also a 5-minute rest between the aerobic exercise session and the low-intensity strength exercise session.

The AER&HST protocol was the same as the AER&HST protocol except for the extra load. The extra load in the AER&HST group was increased to 10% body weight.

### Blood lactic acid level measurement

Blood lactic acid was used to quantify the intensity of exercise. Speed-ramped treadmill running with a load was used to simulate strength exercise. The blood samples were collected through the eye canthus immediately after the first formal training day. The rats in the Sed group were not subjected to any physical exercise. They spent the entire period in their own cages. Blood samples were collected in the same way. Test strips were used to measure the concentration of blood lactic acid with the aid of a Lactate Scout Analyzer (EKF, Cardiff, UK).

### Whole body muscle weight and hind limb muscle weight measurement

The normalized whole body muscle weight and hind limb muscle weight were used to evaluate the effectiveness of strength exercise. The rats were anesthetized with a single intraperitoneal injection of sodium pentobarbital after two months of formal training. The body weight of the rats was measured by an electronic scale. The overall and regional muscle was analyzed with a dual-energy X-ray absorptiometer (DXA) system (Norland XR-800, Fort Atkinson, WI, USA). Then, the percentage of whole body muscle and the percentage of hind limb muscle were calculated for analysis.

### Morris water maze

The Morris water maze was used to evaluate spatial learning and memory. Eight rats from each group were tested in a black water maze tank (1.6 m diameter) 24 hours after exercise. The tank was filled with water (25 ± 2°C) to a depth of approximately 8 cm below the tank rim. The rats were dried with towels and warm air between blocks and before being returned to their home cages.

During the training session, the rats were trained to find the hidden platform, with 4 trials per day for 6 days. A platform was hidden 2 cm below the surface of the water in the center of the 3^rd^ quadrant. The starting points were randomly changed every day. Each trial lasted until the rats had found the platform or for a maximum of 90 s. Rats that failed to locate and climb onto the platform within the 90 s were guided to the platform by the experimenter before being removed from the maze. At the end of each trial, the rats were kept on the platform for 5 s to allow them to remember the location and then dried with a towel and placed under a 37°C lamp between trials. The time to reach the platform (latency) and the swimming speed were recorded automatically by a video tracking system. To measure the rate of acquisition, the latency to reach the platform was averaged over all four trials each day.

During the probe test on the 7^th^ day, the platform was removed. All rats were dropped at the same starting point and allowed to navigate the maze for 90 s. The number of times that the rat swam across the virtual platform and the time spent in the platform quadrant were recorded by the video tracking system.

### Bromodeoxyuridine labeling

Eight rats from each group were intraperitoneally injected with 10 mg/mL bromodeoxyuridine (BrdU) after the Morris water maze test. A total of 4 injections per rat were performed within 12 hours (interval 4 hours between two injections). The dose of BrdU was 50 mg kg^-1^ (total 200 mg kg^-1^) body weight to label dividing cells. Twelve hours after the last BrdU injection, the rats were anesthetized and perfused with PBS and 4% paraformaldehyde (PFA). The brains were immediately dissected and postfixed in 4% PFA at 4°C for 24 hours.

### Tissue processing, immunostaining, confocal imaging, and cell counting

Coronal sections (40 μm thick) were cut through the entire DG of the left or right (randomized) hippocampus with a vibratome (Leica VT 1000 S). All brain sections were collected sequentially in a 6-well plate, which was filled with cryoprotectant solution (30% sucrose + 30% ethylene glycol in 0.1 M phosphate buffer, pH 7.4). The samples were stored at -20°C until staining.

The sections were first incubated with 2 N HCl at room temperature for 1 hour. Thereafter, the sections were neutralized with 0.1 M borate buffer and then incubated with a rat anti-BrdU antibody (1:100, Accurate Chemical and Scientific Corporation, Westbury, NY, USA) at 4°C overnight. After extensive washing, the sections were incubated with a donkey anti-rat secondary antibody conjugated to Alexa 488 (Molecular Probes) at room temperature for 2 hours. Ki67 immunostaining was conducted with a rabbit anti-Ki67 antibody (1:200, Thermo Fisher Scientific, Rockford, IL, USA) and a donkey anti-rabbit secondary antibody conjugated to Alexa 555 (1:200, Thermo Fisher Scientific, Rockford, IL, USA).

Images were captured using an Olympus FV1000 Viewer confocal microscope (Tokyo, Japan). Every 6^th^ section throughout the DG was analyzed for the number of BrdU+ cells and Ki67+ cells, which were counted by an investigator who was blinded to the treatment.

### Real-time PCR

Four rats from each group were sacrificed immediately after the formal training. The brains were removed, and the left hippocampi were collected and frozen in liquid nitrogen for RNA extraction. Total RNA was extracted using Trizol reagent (Invitrogen, Carlsbad, CA, USA) as recommended by the manufacturer. Complementary DNA was synthesized using transcript one-step gDNA removal and cDNA Synthesis SuperMix (AT-311-03, TransGen Biotech, Beijing, China) according to the manufacturer’s protocol. Next, real-time PCR was carried out using SYBR Premix Ex Taq^TM^ II (RR820A, Takara, Dalian, China) with an ABI StepOnePlus Real-Time PCR System. Each sample was assayed in triplicate, and the mRNA level was normalized to the GAPDH mRNA level using the 2^-ΔΔCT^ method.

The following primers were used: BDNF: Forward primer 5’-CAGTATTAGCGAGTGGGTCA-3’, Reverse primer 5’-GATTGGGTAGTTCGGCATT-3’; GAPDH: Forward primer 5’-TGCTGAGTATGTCGTGGAG-3’, Reverse primer 5’-GTCTTCTGAGTGGCAGTGAT-3.

### β-HB measurement

The right hippocampus was used for β**-**HB measurement. β**-**HB levels were measured using a β**-**HB Assay kit (MAK041, Sigma) according to the manufacturer’s protocol. Briefly, the hippocampus was homogenized in 4 volumes of cold β-HB assay buffer and centrifuged at 4°C to remove insoluble material. Then, 50 μl reaction mixes were set up and incubated at room temperature for 30 minutes. The absorbance was measured at 450 nm.

## Statistical analysis

All data represent mean ± the standard error of the mean (s.e.m.). ANOVA was used to determine statistical significance (*p < 0.05, **p < 0.01, and ***p < 0.001). All statistical analyses were performed using GraphPad Prism 6.0.

## Results

### Establishment of the exercise paradigms

**Fig 1:**
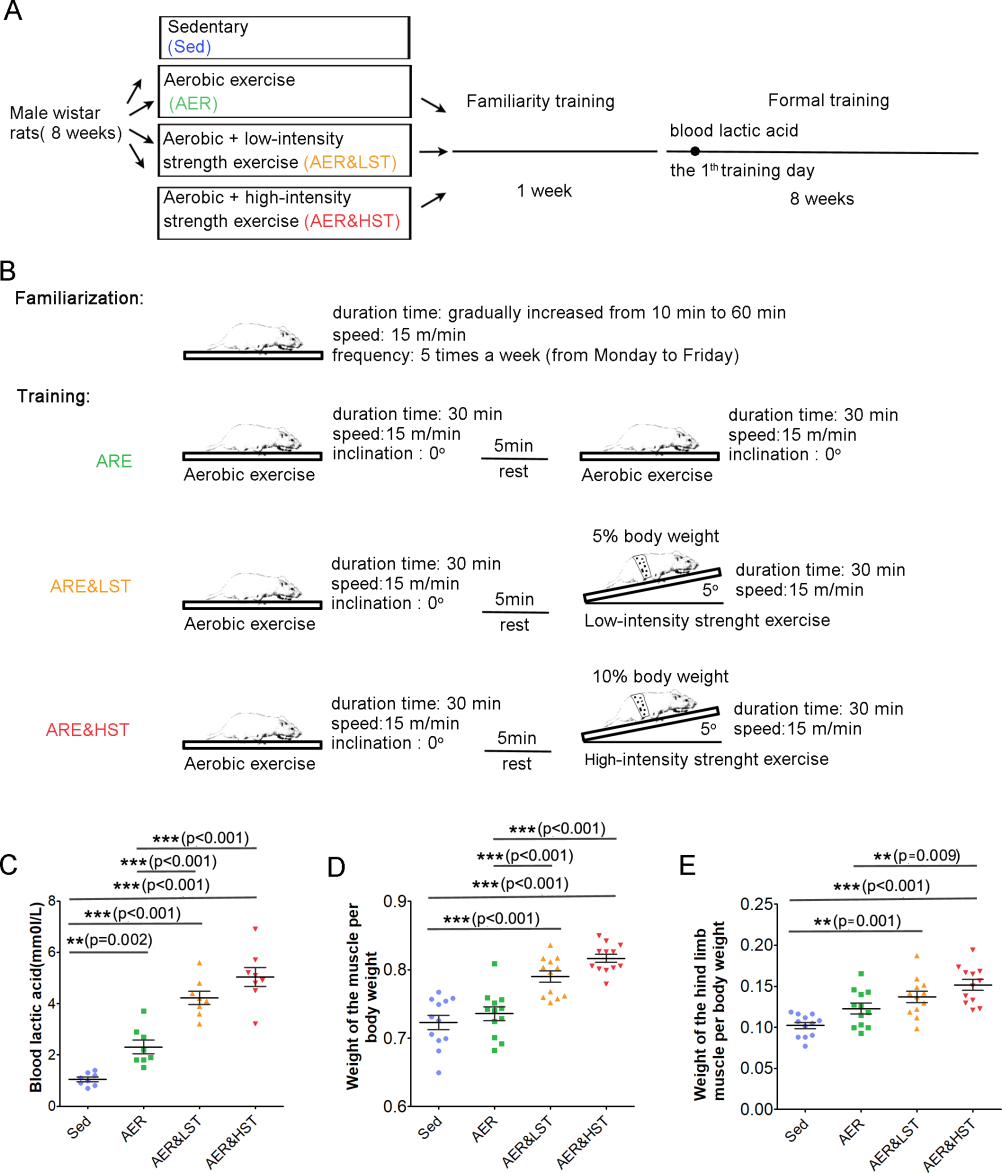
Design of exercise behavior paradigms. Different exercise strategies were implemented in 8-week-old male Wistar rats. (A, n = 8/group) (B). Paradigms of the familiarization exercise strategies. A detailed outline of the training protocol. (C, n = 8). Detection of lactic acid levels in the blood after physical exercise. ***, p < 0.001, **, p < 0.01; one-way ANOVA; n = 8 for (C). Quantitative analysis of whole body muscle weight/body weight (D) and hind limb muscle weight/body weight (E) after two months of training. Data are shown as the mean ± SEM; ***, p < 0.001; **, p < 0.01; one-way ANOVA; n = 8 for (C and D).

Previous studies have shown that aerobic exercise, such as running, improves learning and memory by increasing adult hippocampal neurogenesis in rodents [4, 15]. However, it is still unclear whether aerobic exercise combined with strength exercise will affect the benefits of exercise. To address this question, we designed four training paradigms for adult rats. As shown in Fig 1A, 8-week-old rats were randomly divided into 4 groups: the Sed, AER, AER&LST, and AER&HST groups. All animals received familiarity training for 1 week and then formal training for 8 weeks. During familiarity training, the rats in the AER, AER&LST, and AER&HST groups were trained on the treadmill at a speed of 15 m/min, 5 times per week (from Monday to Friday). The exercise duration gradually increased from 10 minutes to 60 minutes (Fig 1B). After familiarity training, the three exercise groups received formal training for 8 weeks. As shown in Fig 1B, the AER group performed two sessions of aerobic treadmill exercise with a 5-minute rest between the two sessions. For each session, the rats in the AER group ran at a speed of 15 m/min with a 0° inclination for 30 minutes. The AER&LST group performed aerobic exercise for 30 minutes. After 5 minutes of rest, the rats carried out a low-intensity strength exercise for 30 minutes at a speed of 15 m/min and a 5° inclination with a 5% body weight load. In the AER&HST group, the load was increased from 5% body weight to 10% body weight.

Strength exercise is a primarily anaerobic exercise that leads to lactic acid accumulation in the blood. To evaluate the efficiency of our strength exercise paradigms, we first analyzed the level of blood lactic acid immediately after the 1^st^ day of formal training. As shown in Fig 1C, the lactic acid level in the blood was increased in the AER, AER&LST, and AER&HST groups. Moreover, the lactic acid level was much higher in the AER&HST and AER&LST groups than in the AER group, suggesting that the strength exercise paradigms are efficient in increasing the level of blood lactic acid. After two months of training, we further analyzed the percentage of skeletal muscle, which is another parameter associated with strength exercise. As shown in Fig 1D and 1E, the muscle weight of the whole body and the muscle weight of the hind limbs were significantly increased in the strength exercise groups (AER&LST and AER&HST) compared to those of the Sed and AER groups. In addition, the muscle weight of the whole body and the muscle weight of the hind limbs were both significantly increased in the AER&HST group compared to those of the AER&LST group (Fig 1D and 1E). Together, these results indicate that the low- and high-intensity strength exercise paradigms were successfully established.

### Impairment of aerobic exercise-induced cognitive improvements by strength exercise

**Fig 2:**
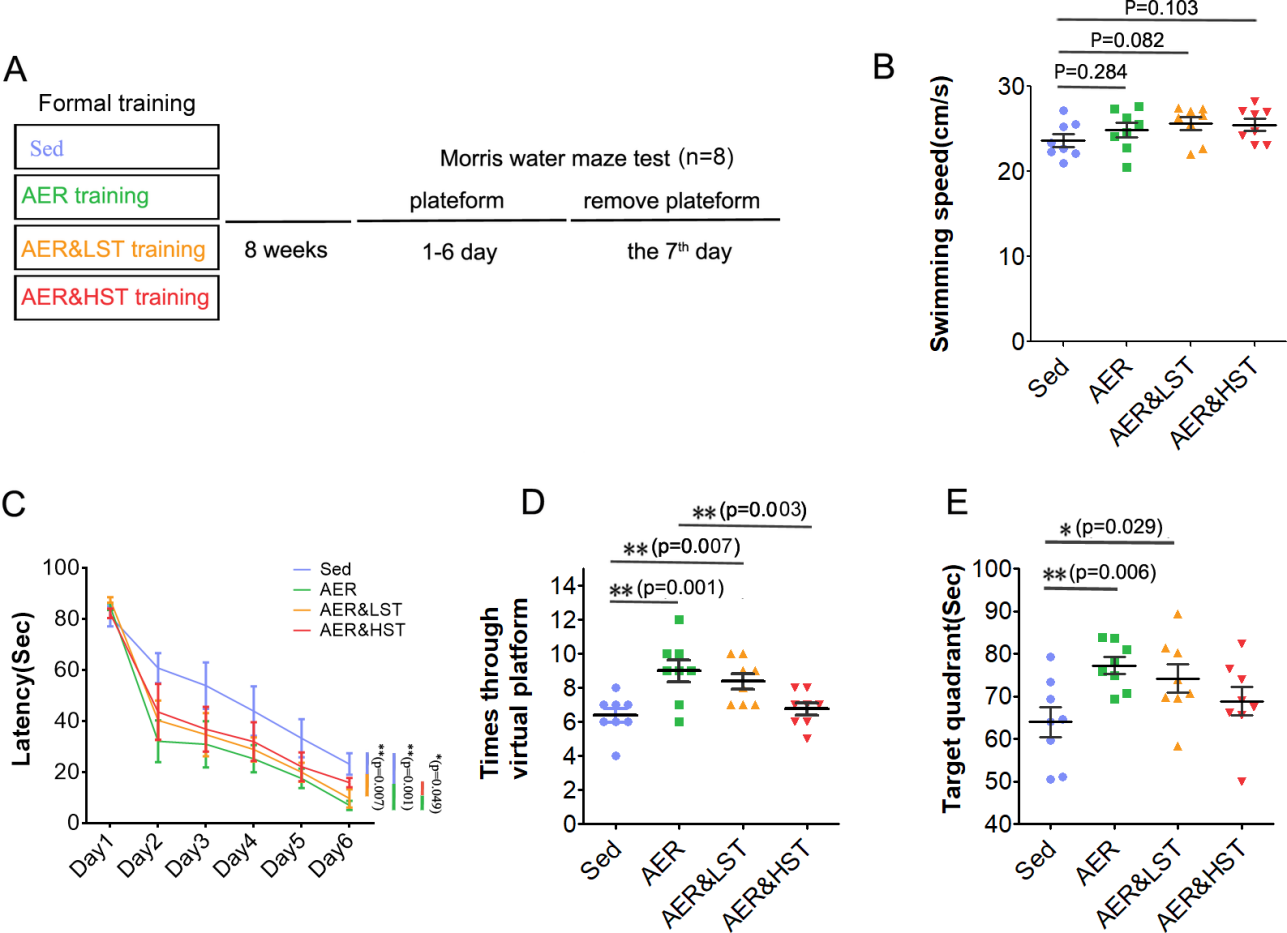
Strength exercise decreases aerobic exercise-induced cognitive function improvements, as indicated by the Morris water maze test. (A). Outline of the experiment. The Morris water maze test was performed after 8 weeks of training. Each group of rats was trained with the hidden platform for 6 days, and on the seventh day, the platform was removed. Quantitative analysis of the swimming speed on the first day (B), the latency to the platform for six days of training (C), the number of platform crosses in the probe test (D), and the time spent in the target quadrant (E). Data are shown as the mean ± SEM; **, p < 0.01, *, p < 0.05; one-way ANOVA; n = 8.

To explore the effect of aerobic exercise combined with strength exercise on cognitive performance, we evaluated the spatial learning and memory of rats by a Morris water maze experiment (Fig 2A). The swimming speed in the exercise groups was similar to that in the Sed group (Fig 2B). As shown in Fig 2C, the rats in the AER group spent less time finding the hidden platform than those in the Sed group, suggesting that aerobic exercise improved spatial learning and memory. However, although the rats in the AER&LST group also spent less time finding the hidden platform than those in the Sed group, little difference was observed between the AER&HST group and the Sed group, suggesting that strength exercise impaired the aerobic exercise-induced improvement in spatial learning and memory. As expected, the AER&HST group spent more time finding the hidden platform than the AER group (Fig 2C). During the probe test, the platform crossing times and the duration in the target quadrant were significantly increased in the AER group compared with those in the Sed group (Fig 2D and 2E). In comparison to those of the AER group, the platform crossing times were decreased by strength exercise in an intensity-dependent manner (Fig 2D), and the duration in the target quadrant was decreased by high-intensity strength exercise (Fig 2E). These results suggest that aerobic exercise improves spatial learning and memory in rats and that this benefit is negatively regulated by strength exercise.

### Impairment of aerobic exercise-induced adult hippocampal neurogenesis by strength exercise

**Fig 3:**
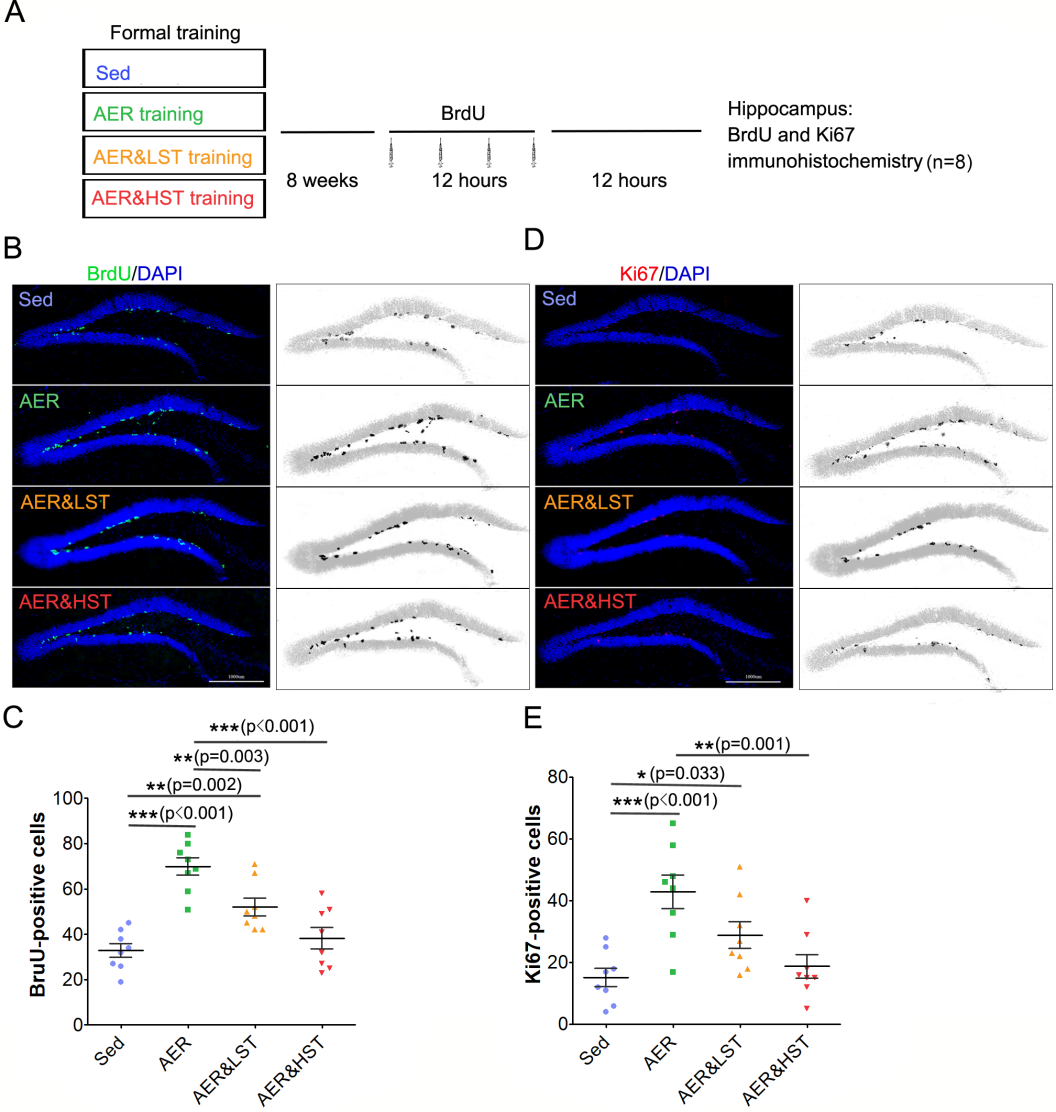
Strength exercise reduces aerobic exercise-induced adult hippocampal neurogenesis. (A). Outline of the experiment. BrdU was injected four times into each group of rats within 12 hours after the indicated training. Twelve hours later, the rats were sacrificed, and immunostaining was performed. Coronal brain sections prepared from the indicated training group were stained with an anti-BrdU antibody (green) to label proliferating cells during 4 injections in the dentate gyrus of the hippocampus. (C). Quantitative analysis of BrdU-positive cells in (B). Coronal brain sections were stained with an anti-Ki67 antibody (red) to label proliferating cells in the dentate gyrus. (E). Quantitative analysis of Ki67-positive cells in (D). Data are shown as the mean ± SEM; ***, p < 0.001, **, p < 0.01; one-way ANOVA; n = 8 for (B); ***, p < 0.001, **, p < 0.01, *, p < 0.05; one-way ANOVA; n = 8 for (C, E).

To investigate whether adult hippocampal neurogenesis is regulated by strength exercise, we examined adult hippocampal neurogenesis in the four groups by BrdU labeling and Ki67 immunostaining. As shown in Fig 3A, 8 rats from each group were intraperitoneally injected with BrdU and sacrificed after the last injection (Fig 3A). In accordance with previous studies, the number of BrdU+ cells was increased in the AER group compared with that in the Sed group [4, 16], supporting the conclusion that aerobic exercise promotes adult hippocampal neurogenesis. Moreover, the number of BrdU+ cells in the AER&LST group was increased compared to that of the Sed group and decreased compared to that of the AER group. In addition, the number of BrdU+ cells in the AER&HST group was decreased compared to that of the AER group and the AER&LST group (Fig 3B and 3C). To further confirm the increased proliferation of adult hippocampal neural stem cells, the brain sections obtained from the four groups were immunostained with an anti-Ki67 antibody, which is a marker for cell proliferation [17]. These results showed an increase in the number of Ki67+ cells in the AER and AER&LST groups compared to that of the Sed group and a decrease in the number of Ki67+ cells in the AER&LST group and a much more significant decrease in the number of Ki67+ cells in the AER&HST group compared to that of the AER group (Fig 3D and 3E). Together, these results indicate that strength exercise impairs aerobic exercise-induced adult hippocampal neurogenesis.

### Suppression of aerobic exercise-induced β-HB synthesis and BDNF transcription by strength exercise

**Fig 4:**
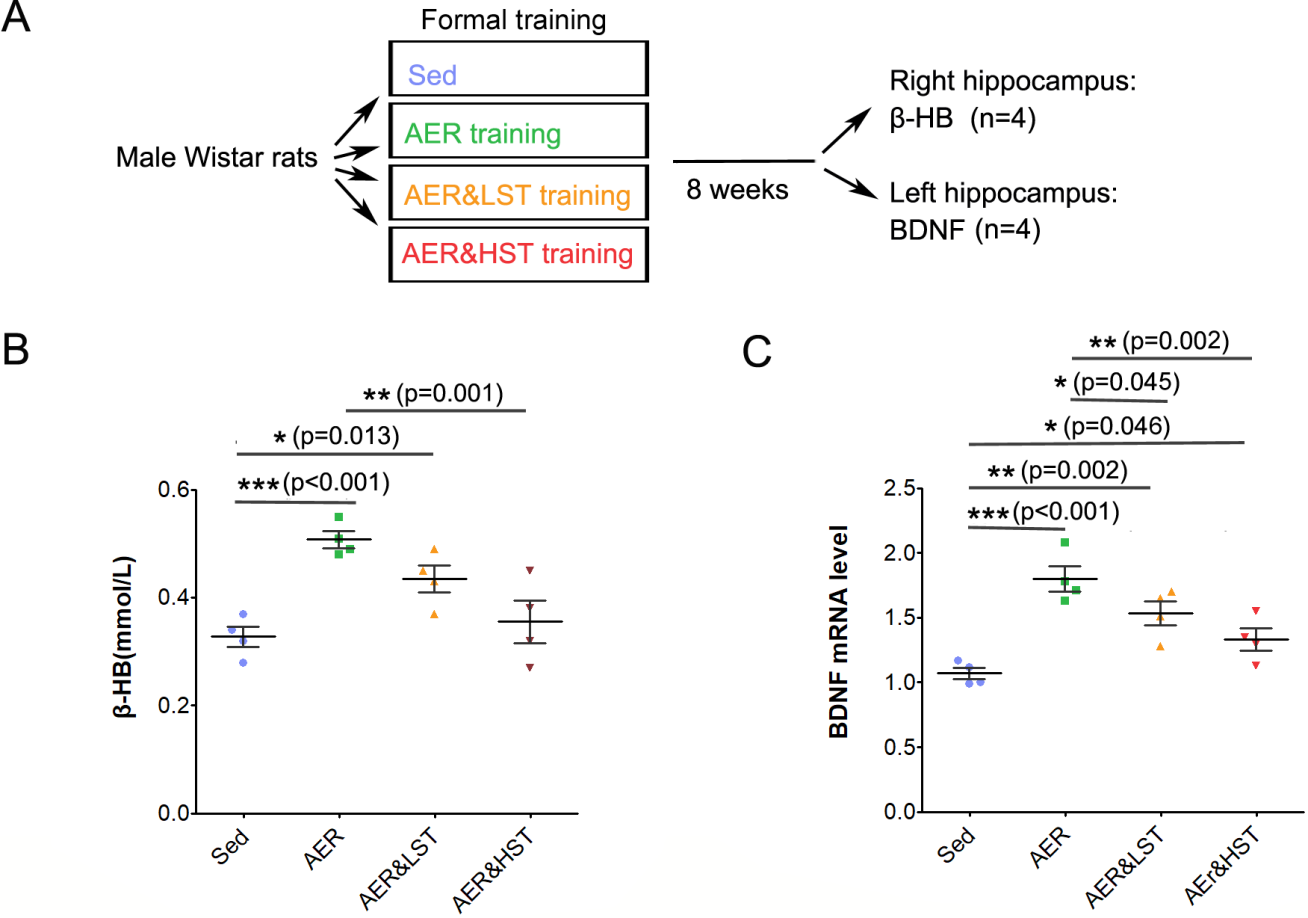
Strength exercise reduces the aerobic exercise-induced increases in the levels of β-HB and BDNF in the hippocampus. (A). Outline of the experiment. The hippocampus was dissected from each group of rats for analysis after training. (B). The β-HB level of each group of rats was analyzed in the right hippocampus. (C). BDNF mRNA levels in each group of rats were determined in the left hippocampus. Data are shown as the mean ± SEM; ***, p < 0.001, **, p < 0.01, *, p < 0.05, one-way ANOVA; n = 4 for (B, C).

How does strength exercise suppress aerobic exercise-induced cognitive performance and adult hippocampal neurogenesis? Considering that aerobic exercise depends on both carbohydrate and fat metabolism and that strength exercise depends on carbohydrate metabolism, we speculated that fat metabolism could play an essential role in this event. In view of the literature, β-HB, an intermediate product of fat metabolism, attracted our attention, as it has been reported to be involved in BDNF expression and neuroprotection [18]. To determine whether strength exercise regulates β-HB and transcription of its downstream effector, BDNF, we analyzed the level of β-HB by spectrophotometry and the transcription of BDNF by real-time PCR. Four rats from each group were sacrificed immediately after the last formal training session, and the hippocampus was dissected for further assay (Fig 4A). Compared with those in the Sed group, the levels of both β-HB and BDNF mRNA were significantly increased in the AER group (Fig 4B and 4C). In comparison to the AER group, the level of β-HB was decreased in the AER&HST group (Fig 4B), and the level of BDNF mRNA was decreased in both the AER&LST group and the AER&HST group (Fig 4C). These results suggest that strength exercise suppresses the aerobic exercise-induced increases in β-HB levels and BDNF expression.

## Discussion

Aerobic exercise combined with strength exercise is composed of both aerobic exercise and strength exercise. The results obtained in our study suggest that aerobic exercise improves cognitive function and promotes adult hippocampal neurogenesis, which is consistent with the findings of previous studies [4, 15, 16]. In the Morris water maze test, the rats in the AER group required less time to find the platform and spent more time in the target quadrant (Fig 3). Adult hippocampal neurogenesis was increased in the AER group, as revealed by BrdU labeling and Ki67 immunostaining (Fig 4). Intriguingly, cognitive function and adult hippocampal neurogenesis were improved in both the AER group and the AER&LST group, but AER&LST exhibited a less significant effect than AER (Figs 3 and 4). Furthermore, the rats in the AER&HST group did not show any aerobic exercise-induced improvements in cognitive function or adult hippocampal neurogenesis (Figs 3 and 4), demonstrating that strength exercise weakened these aerobic exercise-induced benefits in an intensity-dependent manner.

Strength exercise has irreplaceable beneficial effects on the human body, such as increasing muscle strength and bone mineral density, improving neuromuscular performance and controlling body weight and body composition [19–21]. However, the effects of strength exercise on adult hippocampal neurogenesis are controversial. Strength exercise promotes adult hippocampal neural stem cell proliferation, increases the survival of newborn neurons, and enhances neural plasticity [22, 23], but another study indicated that strength training has little influence on adult hippocampal neurogenesis [24]. This controversy does not seem to be due to the training protocols, since both protocols required the animals to climb with 50%, 75%, and 90% of the maximum load. According to our study, strength exercise impairs the aerobic exercise-induced improvements in cognitive function and adult hippocampal neurogenesis. As we did not conduct strength training alone, it is difficult to conclude whether strength exercise contributes to adult hippocampal neurogenesis. However, we want to note that strength exercise together with aerobic exercise is less efficient in inducing neurogenesis than aerobic exercise alone. Strength training may also be beneficial for neurogenesis when compared with the control group under pathological conditions. Hyunjin et al. found that intermittent intense exercise protected against cognitive decline in a manner similar to that observed for moderate exercise in chronically stressed mice, suggesting that the frequency but not the intensity determined the effect of exercise on adult neurogenesis and cognitive protection[25]. Thus, further studies are still needed to determine the effect of strength exercise on adult hippocampal neurogenesis.

How does strength exercise suppress aerobic exercise-induced cognitive performance improvements and adult hippocampal neurogenesis? Based on a comparison of the metabolic pathways involved in aerobic exercise and strength exercise, we speculate that fat metabolism may be the key to this problem. β-HB, which is synthesized in the liver and transported to the body through blood circulation, is an intermediate product of fat metabolism. It is able to pass through the blood-brain barrier and becomes one of the major energy sources in the brain [26–28]. β-HB has neuroprotective effects, which alleviate neurodegenerative disease by improving mitochondrial functions [29, 30]. β-HB also promotes the expression of BDNF, an important neurotrophic factor that is associated with synapse plasticity and adult hippocampal neurogenesis [18, 31]. Intriguingly, fat metabolism is highly dependent on exercise intensity. As exercise intensity increases, lactic acid accumulates in the body, which in turn inhibits fat mobilization [32–34] and reduces the level of β-HB [35]. Our study showed that aerobic exercise increased the levels of β-HB and the BDNF transcript; however, compared with the effects of aerobic exercise, aerobic exercise combined with strength exercise reduced the levels of β-HB and the BDNF transcript (Fig 4). Therefore, we speculate that aerobic exercise improves adult hippocampal neurogenesis and cognitive function, probably through increasing β-HB-induced BDNF transcription. However, strength exercise generates large amounts of lactic acid, which suppresses the production of β-HB, as well as subsequent BDNF transcription and aerobic exercise-induced cognitive improvements. Therefore, strength exercise intensity is an important factor in determining the effects of aerobic combined with strength exercise. If exercising at a proper intensity, aerobic exercise combined with strength exercise may also exert good effects on adult neurogenesis in the hippocampus.

## Conclusions

1. Strength exercise impairs aerobic exercise-induced cognitive function improvements.
2. Strength exercise reduces aerobic exercise-induced adult hippocampal neurogenesis.

## Supporting information

S1 Table Raw data for all figures. (DOCX)
