# Supplementary material for: Strength exercise weakens aerobic exercise-induced cognitive improvements in rats
Source: PLoS One. 2018 Oct 10;13(10):e0205562. doi: 10.1371/journal.pone.0205562 (PMC6179267; doi:10.1371/journal.pone.0205562)
Supplement: S1 Table — (DOCX) [file pone.0205562.s001.docx]

**Supporting Information**

Fig 1 C Blood Lactic acid (mmol/L, X±SEM, n=8)

|  | Sed | AER | AER&LST | AER&HST |
| --- | --- | --- | --- | --- |
| Blood Lactic  acid | 1.05±0.09 | 2.31±0.26 | 4.23±0.26 | 4.94±0.41 |

Fig 1 D Weight of the muscle per body weight ( X±SEM, n=12)

|  | Sed | AER | AER&LST | AER&HST |
| --- | --- | --- | --- | --- |
| Weight of the muscle per body weight | 0.72±0.04 | 0.74±0.03 | 0.79±0.03 | 0.82±0.02 |

Fig 1 E Weight of the hind limb muscle per body weight ( X±SEM, n=12)

|  | Sed | AER | AER&LST | AER&HST |
| --- | --- | --- | --- | --- |
| Weight of the hind limb muscle per body weight | 0.10±0.01 | 0.12±0.02 | 0.14±0.02 | 0.15±0.02 |

Fig 2 B Swimming speed (cm/s, X±SEM, n=8)

|  | Sed | AER | AER&LST | AER&HST |
| --- | --- | --- | --- | --- |
| Swimming  speed | 23.69±0.75 | 24.88±0.85 | 25.65±0.75 | 25.52±0.39 |

Fig 2 C Latency (Sec, X±SEM, n=8)

| Latency | Sed | AER | AER&LST | AER&HST |
| --- | --- | --- | --- | --- |
| Day1 | 81.11±3.95 | 84.85±1.30 | 87.46±1.06 | 82.24±1.87 |
| Day2 | 60.80±5.87 | 32.13±8.19 | 40.34±7.69 | 43.58±10.96 |
| Day3 | 53.92±9.10 | 30.92±9.05 | 34.68±8.47 | 36.83±8.78 |
| Day4 | 43.91±9.71 | 25.24±5.31 | 28.90±4.73 | 31.96±7.60 |
| Day5 | 33.27±7.44 | 17.55±3.80 | 19.97±3.73 | 22.10±5.64 |
| Day6 | 23.17±4.21 | 8.75±2.90 | 9.70±3.56 | 14.66±3.57 |

Fig 2 D Times through virtual plateform (X±SEM, n=8)

|  | Sed | AER | AER&LST | AER&HST |
| --- | --- | --- | --- | --- |
| Times through virtual plateform | 6.38±0.42 | 9.00±0.65 | 8.38±0.46 | 6.75±0.37 |

Fig 2 E Target quadrant (Sec, X±SEM, n=8)

|  | Sed | AER | AER&LST | AER&HST |
| --- | --- | --- | --- | --- |
| Target  quadrant | 63.98±3.59 | 77.25±1.96 | 74.2±3.33 | 68.88±3.39 |

Fig 3 C BrdU-positive cells ( X±SEM, n=8)

|  | Sed | AER | AER&LST | AER&HST |
| --- | --- | --- | --- | --- |
| BrdU-positive  cells | 32.88±3.08 | 69.88±3.86 | 52.13±3.91 | 36.43±5.03 |

Fig 3 E Ki67-positive cells ( X±SEM, n=8)

|  | Sed | AER | AER&LST | AER&HST |
| --- | --- | --- | --- | --- |
| Ki67-positive  cells | 15.13±3.01 | 42.88±5.45 | 28.88±12.19 | 19.29±4.40 |

Fig 4 B β-HB (mmol/L, X±SEM, n=4)

|  | Sed | AER | AER&LST | AER&HST |
| --- | --- | --- | --- | --- |
| β-HB | 0.33±0.02 | 0.51±0.02 | 0.44±0.03 | 0.36±0.04 |

Fig 4 C BDNF mRNA level (mmol/L, X±SEM, n=4)

|  | Sed | AER | AER&LST | AER&HST |
| --- | --- | --- | --- | --- |
| BDNF mRNA  level | 1.07±0.04 | 1.8±0.10 | 1.54±0.10 | 1.33±0.09 |
